# Clinical characteristics and long-term prognosis of talaromycosis in children with novel and reported inborn errors of immunity

**DOI:** 10.3389/fimmu.2026.1834635

**Published:** 2026-05-13

**Authors:** Qian Lu, Xianghui Li, Zhiwen Jiang, Tiantian Li, Bingkun Li, Lan Huang, Qihua Huang, Dongmei Hu, Chunying Lv, Guoqun Liu, Jialing Zhong, Jingjing Lin, Liuwei Liao, Qianfeng Qin, Sha Qin, Haotian Shao, Zhiyi Wang, Xiuying Li, Li Jiang, Xinyu Zhang, Lili Wei, Jiarong Liang, Dongyan Zheng, Shuangjie Wang, Weixuan Wu, Kaisu Pan, Yanqing Zheng, Yanning Li, Qing Tang, Min Jiang, Wanqing Liao, Cunwei Cao

**Affiliations:** 1 Department of Dermatology and Venereology, the First Affiliated Hospital of Guangxi Medical University, Nanning, China; 2 Guangxi Scientific and Technological Innovation Cooperation Base of Mycosis Prevention and Control, Nanning, China; 3 Guangxi Key Laboratory of Mycosis Prevention and Treatment, Nanning, Guangxi, China; 4 Fangchenggang Wanqing Institute of Mycosis Prevention and Control, Fangchenggang, China; 5 School of Public Health Guangxi Medical University, Nanning, China; 6 Department of Pediatrics, The First Affiliated Hospital of Guangxi Medical University, Nanning, China; 7 Shanghai Key Laboratory of Molecular Medical Mycology, Shanghai Changzheng Hospital, Naval Medical University, Shanghai, China

**Keywords:** children, HIV-uninfected, inborn errors of immunity, management, prognosis, talaromycosis

## Abstract

**Importance:**

Little is known about the long-term prognosis of patients with talaromycosis and inborn errors of immunity (IEI).

**Objective:**

We aimed to evaluate the clinical characteristics and long-term outcomes of talaromycosis in children with IEI on a 13-year cohort study.

**Main outcomes and measures:**

An observational study was conducted on pediatric patients with talaromycosis at tertiary hospitals in southern China during 2012 to 2024. Data on demographic information, clinical features, immunological characteristics, genetic tests, antifungal treatment, and long-term prognosis were collected for analysis.

**Results:**

Among the 329 patients with talaromycosis without HIV, children accounted for 7%. 100% children with talaromycosis were diagnosed as IEI, and a total of 18 children were finally enrolled. All children were identified with IEI, including five novel genetic defects (*IL7R*, *LYST*, *PLCG2*, *DOCK8*, and *KRAS* deficiency) and five reported genetic defects (STAT1-GOF, *IL2RG*, *CD40L*, STAT3-LOF, *CARD9* deficiency). Most children exhibited decreased lymphocytes, NK cells, and immunoglobulin levels. Half of children had been suffered from severe complications, such as sepsis or septic shock, thus had to received advanced life support in ICU. The median antifungal treatment duration was 16 months (IQR: 0.8-72.5 months). Amphotericin B and voriconazole are commonly used for induction therapy. Voriconazole and itraconazole are commonly used for maintenance therapy. Within 2 weeks of induction antifungal treatment, 3 patients died, and another 3 patients died within 24 weeks. At the end of observation, despite one child died of leukemia and one lost to follow-up, 4 achieved long-term relapse-free survival without antifungal treatment, and 6 were stable with maintenance antifungal therapy (One survived for more than 20 years, the longest recorded survival now). Most children benefited from immune boosting therapy, including 2 received HSCT intervention.

**Conclusions and relevance:**

Talaromycosis serves as an early warning indicator for IEI in HIV-uninfected children. Treatment and long-time management remain a challenge. Besides, long lasting anti-fungal and immune boosting therapy may be necessary, while HSCT intervention could provide potential benefits.

## 1 Introduction

Talaromycosis caused by *Talaromyces marneffei* (*T. marneffei*) infection is an invasive mycosis that is endemic in Southeast Asia and Southern China (1). It is estimated that approximately 17,300 cases of talaromycosis are diagnosed annually, with mortality rates up to 33% due to typically fatal disseminated infections (2). By 2022, over 288,000 talaromycosis cases had been reported in 34 countries (3).

Talaromycosis is believed to be closely associated with immunodeficiency, such as acquired immunodeficiency syndrome (AIDS) (1). Anti-IFN-γ autoantibody (AIGA), another acquired immunodeficiency, represent a major risk factor for talaromycosis in HIV-uninfected adult individuals (4). However, whether the inborn immunodeficiency affects the susceptibility towards talaromycosis remained poorly understood (5). Sporadic talaromycosis cases emerged in HIV-uninfected children with Inborn Errors of Immunity (IEI), indicating the potential correlation between IEI and talaromycosis (6).

IEI is a group of genetically determined disorders that result in defects in the development, function, or regulation of the immune system (6). Fungal infectious diseases in children and young adults often indicate IEI. Thus far, 50 cases of IEI-associated talaromycosis have been reported globally. 94% cases were pediatric patients, and the most frequently identified genetic defects include X-linked hyper immunoglobulin M syndrome (XHIM), signal transduction and activator of transcription 3-loss of function (STAT3-LOF), and signal transduction and activator of transcription 1-gain of function (STAT1-GOF) (7). However, due to the lack of knowledge about the potential susceptibility of diverse IEI genetic defects, clinical characteristics and the long-term prognosis, the management of children with talaromycosis remains a threaten challenge.

Therefore, we conducted a 13-year cohort study to explore the relationship between talaromycosis and IEI, as well as analyze the clinical characteristics and long-term outcomes of these patients. These findings may contribute to establish a foundation for future guidelines that address this neglected population.

## 2 Materials and methods

### 2.1 Ethical statement

All study procedures were reviewed and approved by the institutional ethics review board at the First Affiliated Hospital of Guangxi Medical University (2012KY074 and 2015KY146). Written informed consent was obtained from the legal guardians of all pediatric patients.

### 2.2 Study design and population

This multi-center, ambispective cohort study was conducted at three tertiary hospitals from January 2012 to December 2024. This study included patients aged <14 years who were diagnosed with talaromycosis. Diagnosis of talaromycosis: Clinical manifestations consistent with *T. marneffei* infection, and met one of the following criteria: (1) Positive cultures for *T. marneffei* from any clinical specimens, were characterized by dimorphic fungi that grew as a mold at 25°C and produced a soluble red pigment that diffuses into the agar, and grew as a yeast at 37°C. (2) Positive histopathology report (an oval, elongated, sausage-like dividing yeast cell with a transverse septum). (3) Positive molecular findings on qPCR or metagenomic next-generation sequencing (mNGS).

### 2.3 Identification of IEI

The diagnosis of IEIs was based on clinical characteristics and genetic tests, according to the updated classification by the Human Inborn Errors of Immunity Committee of the International Union of Immunological Societies (IUIS) (8). Whole exome gene testing was performed by Illumina sequencing technology (Beijing Maikino Company). The sequencing platform is Illumina HiSeq 2000. The predicted pathogenicity of novel variants was evaluated based on the American College of Medical Genetics and Genomics (ACMG) criteria (9).

### 2.4 Peripheral immunological evaluation

Routine blood test, serum biochemical test, and peripheral immunological test were conducted in all patients to evaluate the condition before treatment. Data were collected from the medical reports. The data included demographic information, domiciles, medical history, clinical manifestations, co-infections, peripheral immunological findings, genetic tests, complications, treatment, and long-term prognoses. Immunoglobulin testing is performed using immunoturbidimetry, while lymphocyte subset testing is performed using flow cytometry. Cytokines were detected by Luminex technology (Shanghai Youning Wei, kit brand: BIO-RAD). Moreover, we evaluated the levels of various of baseline cytokines from 13 children.

### 2.5 Treatment administration

Generally, antifungal treatment included initial (induction) phase and maintenance phase. The induction therapy regimen mainly involved amphotericin B and voriconazole, usually for 2 weeks. Then, they received voriconazole or itraconazole as the maintenance therapy. Antifungal agent selection was based on patient tolerance and financial considerations. In addition, the patients also received IVIg and thymosin as immunotherapy. As a promising treatment for IEI, hematopoietic stem cell transplantation (HSCT) has also been explored in some patients. In the absence of guidelines for treatment in children with talaromycosis and IEI, all treatments were administered based on the clinical experience of the attending physicians.

### 2.6 Follow-up assessment

Patients were followed up at 2 weeks, 24 weeks, and 2 years after initial treatment, and thereafter at least once every 12 months. Patients with active disease received additional follow-up as needed. Follow-up evaluations were categorized as: (1) survival, (2) death, or (3) lost to follow-up.

### 2.7 Data collection and statistical analysis

All data were collected using a standardized form that was based entirely on the medical reports of each patient. Statistical analyses were performed using IBM SPSS version 26. Data were presented as the frequency (%), or median (interquartile range, IQR). The Wilcoxon signed rank test was used to compare the immunological detection indicators of patients before and after treatment; Comparison of cytokine levels between groups using Mann Whitney U test.

## 3 Results

### 3.1 Demographic and clinical characteristics

**Figure 1:**
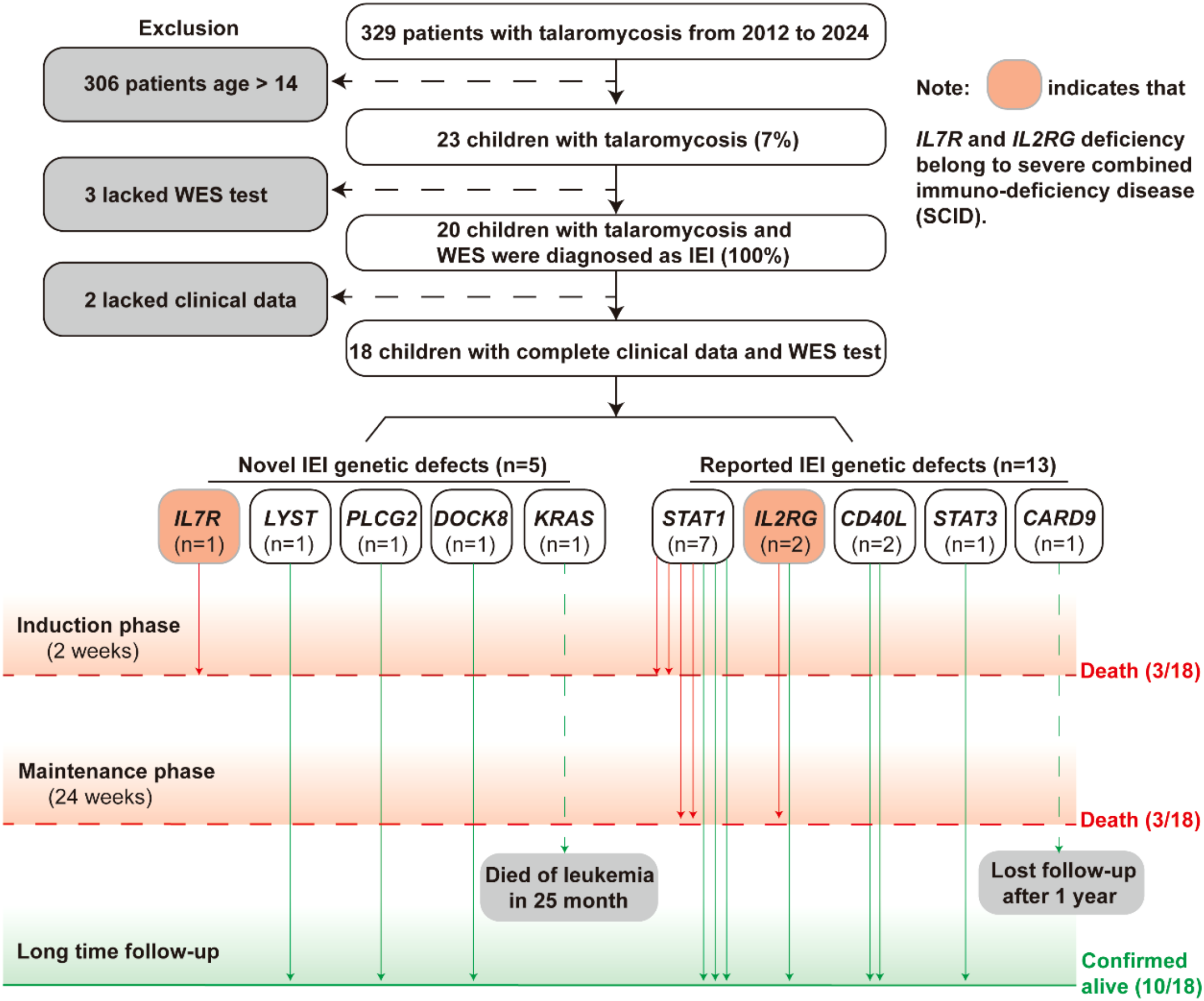
Flow diagram of evaluations of talaromycosis in children with IEI.

**Figure 2:**
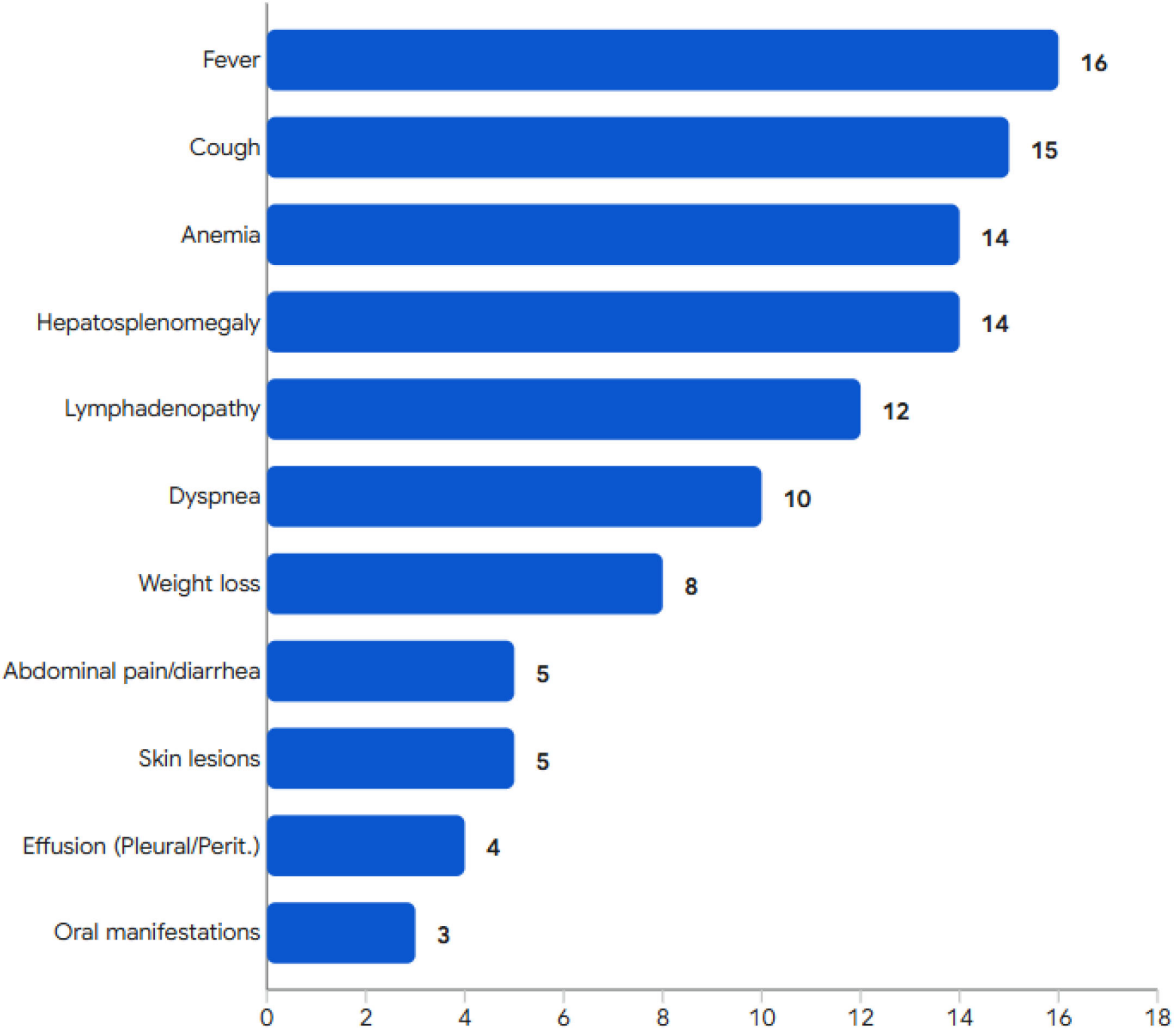
Graphical representation is done for clinical manifestations.

23 pediatric patients (nearly 7%) among 329 cases of HIV-uninfected talaromycosis were diagnosed between January 2012 and December 2024 in our cohort. Among the 23 children, 2 had incomplete clinical data, 3 lacked whole exome sequencing test (WES). Notably, all the 20 children with HIV-uninfected talaromycosis who performed WES were diagnosed as IEI (100%). A total of 18 children were finally enrolled (**Figure 1**), including 7 boys and 11 girls, whose median age at onset of talaromycosis was 20.5 months (IQR 5.8–53.7 months). The most common clinical manifestations included fever, anemia, cough, hepatosplenomegaly, and lymphadenopathy (**Table 1**). Bacille Calmette-Guérin disease occurred in a child (IEI: *IL2RG* deficiency), characteristics as localized skin redness, swelling, ulceration, and pus discharge. Another child with *IL2RG* mutations exhibited positivity for autoantibody (anti-Ro-52 antibody). Due to atypical manifestations, 11 (61.1%) patients were initially misdiagnosed with bronchial pneumonia or tuberculosis. *T. marneffei* was mainly isolated from blood and bone marrow, other sources included BALF, sputum, lymph node, cerebrospinal fluid, and stool. In addition, one child was also diagnosed based on histopathological examination of liver and intestine tissues. The median time from symptom onset to diagnosis was 40 days (IQR 26.5–78 days). The common complications included sepsis or septic shock, severe pneumonia and hemophagocytic lymphohistiocytosis (HLH). In addition, 12 (66.7%) patients were co-infected with other pathogens, including *Cryptococcus*, *Salmonella*, *Cytomegalovirus*, *Pneumocystis jirovecii*, and *Streptococcus pneumoniae*. The clinical characteristics of IEI with talaromycosis are summarized in **Table 1**; **Figure 2**.

**Table 1:** Baseline clinical features of children with talaromycosis and IEI.

ID	Onset age	Sex	Symptoms/signs	Medical history	Infections	Antifungal treatment	Complications	Genetic defect	Follow-up (month)	Outcome
1	6y11m	F	Fever, cough, anemia, lymphadenopathy, hepatosplenomegaly, skin lesions	Iron deficiency anemia, pneumonia	TM (bone marrow, blood), *C. albicans, S. aureus*	VCZ(20m) +ITZ (26m)	–	STAT1	93m	RFS
2	4y2m	F	Fever, cough, weight loss, anemia, dyspnea, lymphadenopathy, hepatosplenomegaly,	CMC	TM (bone marrow, blood, sputum) EBV, *C. albicans*, *M. Pneumoniae*	VCZ (3d)	Sepsis, septic shock, severe pneumonia, HLH, COPD, MODS	STAT1	0.1m	Death
3	4y4m	M	Fever, cough, hematochezia, anemia, dyspnea, lymphadenopathy, hepatosplenomegaly, skin lesions	–	TM (bone marrow, blood, sputum) *A. fumigatus*, *S. aureus, K. pneumoniae*, *C. albicans*, EBV, *P. aeruginosa*	AmB (15d)+VCZ (18d)	Sepsis, septic shock, severe pneumonia, HLH, DIC, ARDS	STAT1	1.5m	Death
4	1y10m	F	Fever, cough, wheezing, lymphadenopathy	Severe pneumonia, mycotic stomatitis	TM (bone marrow, blood), *S. aureus*, EBV, CMV, *H.influenzae*	AmB (10d)+ITZ (54m)	Sepsis, septic shock	STAT1	55m	SRAT
5	1y2m	M	Fever, cough, anemia, dyspnea, pleural and peritoneal effusion, lymphadenopathy, hepatosplenomegaly	–	TM (bone marrow, blood)	AmB (2d)	Sepsis, septic shock, severe pneumonia, HLH, DIC, MODS	STAT1	0.1m	Death
6	7y	F	Fever, anemia, fatigue, lymphadenopathy, splenomegaly, skin lesions	CMC	TM (lymph node)	FLZ (12m)+ITZ#	–	STAT1	53m	SRAT
7	4y11m	M	Fever, cough, anemia, dyspnea, lymphadenopathy, hepatosplenomegaly	Sepsis, pneumonia	TM (blood)	N	Sepsis, septic shock, severe pneumonia, HLH, MODS	STAT1	0.1m	Death
8	4m	F	Fever, cough, weight loss, anemia, abdominal pain and diarrhea, lymphadenopathy, hepatosplenomegaly	G6PD deficiency; perianal abscess	TM (bone marrow, blood)	ITZ(95m)	Respiratory failure	CD40LG	104m	SRAT
9	1y7m	F	Fever, cough, weight loss, anemia, abdominal pain, lymphadenopathy, hepatosplenomegaly	Chronic viral hepatitis B	TM (bone marrow, blood) HBV	VCZ(6m) +ITZ (67m)	Sepsis, HLH	CD40LG	73m	SRAT
10	2y9m	F	Fever, weight loss, nausea, anemia, abdominal pain and diarrhea, lymphadenopathy, hepatosplenomegaly	Crohn’s disease, pneumonia	TM (bone marrow, blood, liver tissue, intestinal tissue) *Oidium tropioale*	VCZ(11m) +ITZ (65m)	–	STAT3	82m	RFS
11	2y4m	M	Fever, cough, weight loss, anemia, dyspnea, abdominal pain and diarrhea, pleural and peritoneal effusion, hepatosplenomegaly	White matter demyelination, hydrocephalus	TM (bone marrow, blood) EBV, CMV *M. Pneumoniae*	VCZ(14m)	Sepsis, HLH, DIC, ARDS, MODS	CARD9	19m	Lost to follow-up
12	2m	M	Fever, cough, anemia, dyspnea, skin lesions, hepatosplenomegaly	Congenital heart diseases (ASD), mycotic stomatitis	TM (blood) *C. Albicans*, HSV, *human Staphylococcus* *subspecies*	VCZ(0.5m)	Severe pneumonia, respiratory failure	IL7R	0.5m	Death
13	2y10m	M	Fever, cough, weight loss, anemia, abdominal pain, oral pain skin lesions, lymphadenopathy	Naso sinusitis	TM (blood, sputum)	VCZ(14m) +ITZ (19m)	–	LYST	132m	RFS
14	4m	F	Fever, cough, weight loss, anemia, dyspnea, cyanosis, hepatomegaly	–	TM (blood, BALF) RSV, Rubella virus, CMV, *A. baumannii*	VCZ(1m)	Severe pneumonia, ARDS	IL2RG	1m	Death
15	6m	F	Fever, nausea, vomiting, anemia, dyspnea, pleural and peritoneal effusion, lymphadenopathy, hepatosplenomegaly	–	TM (bone marrow, blood, BALF, sputum, stool, CSF) *P. jirovecii*, *M. chelonei*, Adenovirus, CMV	AmB (32d)+VCZ (2m)+PCZ(12m)	Severe pneumonia, respiratory failure, MODS	IL2RG	15m	SRAT
16	1y	F	Fever, face pale, cough, weight loss, anemia, dyspnea, pleural and peritoneal effusion, hepatosplenomegaly	Cholecystolithiasis, JMML, Hirschsprung’s disease	TM (bone marrow)	VCZ(6m) +ITZ (1m)	Sepsis, septic shock, severe pneumonia, HLH, COPD, MODS	KRAS	25m	Death*
17	6m	M	Cough, dyspnea	–	TM (BALF) *H.influenzae*, *P. jirovecii*, *A. fumigatus*	VCZ(10m) +ITZ (19m)	–	DOCK8	29m	SRAT
18	3m	F	Cough, wheezing, anemia	mycotic stomatitis	TM (BALF) *P. jirovecii*, *A. fumigatus*, CMV, *C. albicans*	ITZ (3m)	–	PLCG2	13m	RFS

CMC, chronic mucocutaneous candidiasis; G6PD, glucose-6-phosphate dehydrogenase; ASD, atrial septal defect; JMML, juvenile myelomonocytic leukemia; TM, *Talaromyces marneffei*; EBV, Epstein Barr virus; HBV, hepatitis B virus; HSV, herpes simplex virus; RSV, respiratory syncytial virus; BALF, bronchoalveolar lavage fluid; CSF, cerebrospinal fluid; AmB, amphotericin B; VCZ, voriconazole; ITZ, itraconazole; FLZ, fluconazole; PCZ, posaconazole; HLH, hemophagocytic lymphohistiocytosis; COPD, chronic obstructive pulmonary disease; MODS, multiple organ dysfunction syndrome; ARDS, acute respiratory distress syndrome; DIC, disseminated intravascular coagulation; SRAT, stable with regular antifungal therapy; RFS, relapse-free survival without antifungal treatment; # the duration of antifungal treatment is unknown, N not receiving antifungal treatment, * the patient died of other diseases. – means no complications occurred in that patient. Y, M, and d mean year (s), month (s) and day (s) in onset age and antifungal treatment.

### 3.2 Peripheral immunological evaluation

The peripheral immunological profiles of the children are summarized in **Table 2**. All patients were HIV negative. In summary, decreased lymphocytes and immunoglobulin levels are common features at baseline. Among the children studied, 87.5% (14/16) of the patients had markedly decreased NK cells. 64.3% (9/14) presented decreasing T lymphocyte counts, among these children, 57.1% (8/14) presented decreasing CD4+ T cells and 46.1% (6/13) presented decreasing CD8+ T cells, and CD4+/CD8+ T cell ratios exhibited a decrease in 38.5% (5/13) patients. More than half of them exhibited low levels of IgA. 40.0% (6/15) of the children were found to have low complement C3, with five of these cases also exhibiting simultaneously low complement C4 levels.

**Table 2:** Laboratory assays about immunity of children with talaromycosis and IEI.

Immunoassays findings	Reference range	Baseline	End of observation	P value
Lymphocyte subsets				
T lymphocyte cell (%)	50-80	60.6 (51.7-69.0)	67.7 (53.3-74.6)	P>0.05
CD4+ subsets cell (%)	30-50	26.0 (18.9-35.4)	35.1 (28.7-39.8)	P>0.05
CD8+ subsets cell (%)	20-40	20.7 (14.2-30.6)	22.4 (18.5-30.7)	P>0.05
The ratio of CD4/CD8	1.0-2.0	1.3 (0.6-2.2)	1.4 (0.9-1.9)	P>0.05
NK cell (%)	5-20	2.4 (0.8-3.6)	6.2 (3.8-29.7)	P>0.05
Immunoglobulin				
IgG (g/L)	6.6-17.5	7.1 (4.9-10.9)	9.8 (6.1-14.2)	P<0.05
IgA (g/L)	0.06-2.21	0.5 (0.2-0.9)	0.6 (0.3-1.3)	P>0.05
IgM (g/L)	0.01-3.14	0.6 (0.3-0.9)	0.8 (0.4-1.0)	P>0.05
Complements C3 (g/L)	0.9-1.8	1.0 (0.5-1.2)	–	–
Complements C4 (g/L)	0.1-0.4	0.3 (0.2-0.4)	–	–

Means the parents’ results were not available. End of observation means that data were collected at the last follow-up time point before this manuscript was wrote. Data were displayed as median (IQR).

**Figure 3:**
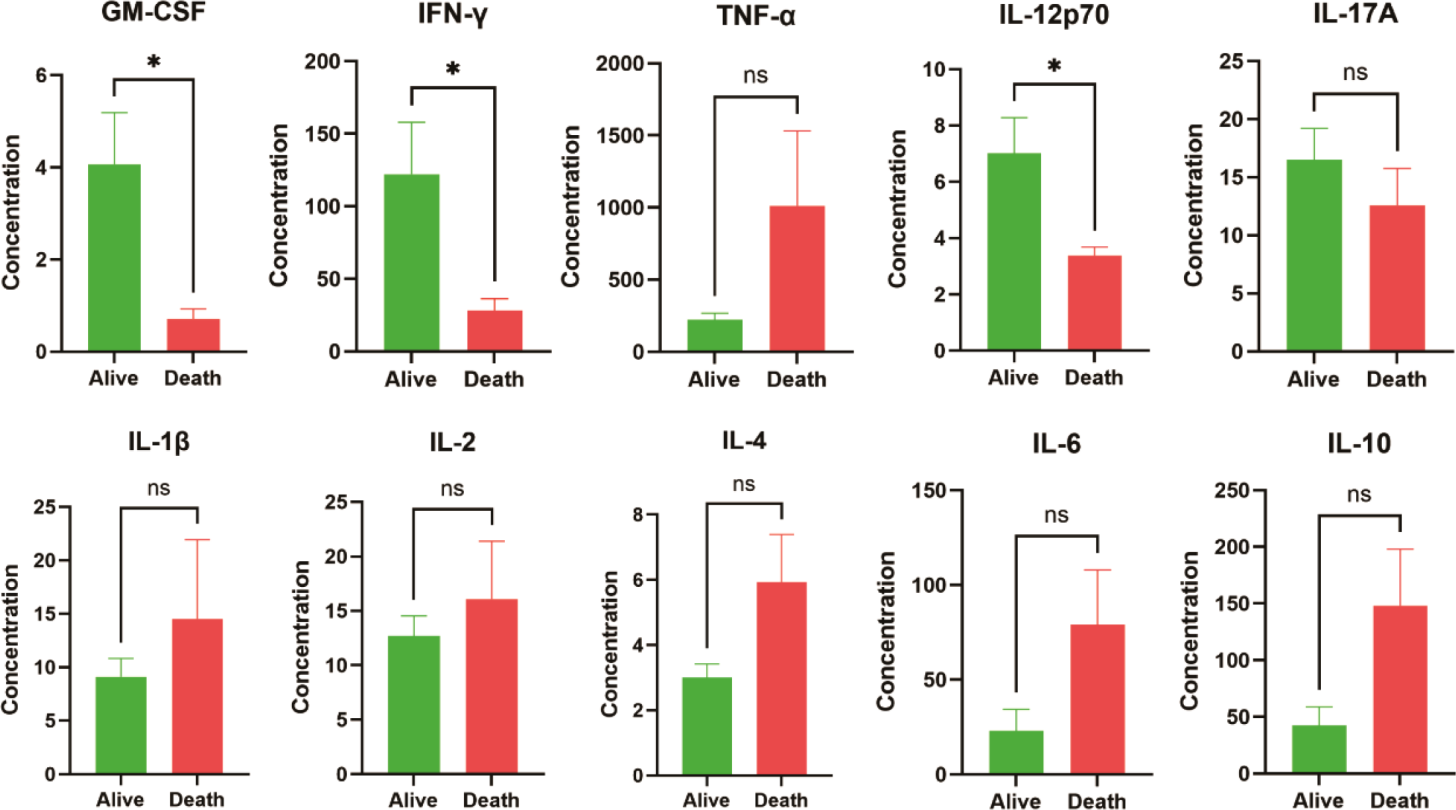
Baseline cytokine evaluations of talaromycosis in children with IEI (Alive vs. Death). * means p<0.05, ns means not significant.

Baseline cytokines were further tested in 13 children, ultimately 9 children were alive and 4 died (IEI: 3 STAT1-GOF and 1 *IL2RG* deficiency) during the follow-up (**Figure 3**). Compared with the alive children, died children exhibited significantly lower levels of GM-CSF, IFN-γ, and IL-12p70. Additionally, cytokine levels including TNF-α, IL-1β, IL-2, IL-4, IL-6, and IL10 of died children were higher, possibly link to the compensatory cytokine storm.

### 3.3 Genetic mutation and inborn errors of immunity

WES was performed to identify IEI. Gain-of-function (GOF) mutations in *STAT1* resulted in STAT1-GOF in seven children. Three children were diagnosed with SCID caused by *IL2RG* or *IL7R* mutations. Two children were confirmed to have hyper IgM syndrome (HIGM) due to mutations in the *CD40L* gene. Two children were diagnosed with hyper immunoglobulin E syndrome (HIES) due to mutations in STAT3-LOF or *DOCK8* genes. One child was confirmed to have Chédiak-Higashi Syndrome (CHS) due to mutations in the *LYST* gene. One child with a *KRAS* gene mutation was diagnosed with *RAS*-associated Autoimmune Leukoproliferative Disease (RALD), and one child with a *PLCG2* gene mutation was diagnosed with an Autoinflammatory Disease (AID). The child who lost follow up in 1 year was diagnosed with *CARD9* deficiency due to compound heterozygous mutations in the *CARD9* gene. The characteristics of IEI are summarized in **Table 3**.

**Table 3:** Genetic mutations in children with talaromycosis and IEI.

Patient	Genetic defect	Nucleotide variation	Types of gene mutation	Protein consequence	Inheritance	Previously reported	Pathogenicity (ACMG classification)
P1	STAT1	c.821G>A(exon10)	Nonsynonymous Mutation	p.Arg274Gln	AR	Yes	Pathogenic
P2	STAT1	c.1175T>C(exon14)	Nonsynonymous Mutation	p.Met392Thr	novel mutation	No	Pathogenic
P3	STAT1	c.820C>T(exon10)	Nonsynonymous Mutation	p.Arg274Trp	AD	Yes	Likely pathogenic
P4	STAT1	c.605T>C(exon8)	Nonsynonymous Mutation	p.Met202Thr	novel mutation	No	Likely pathogenic
P5	STAT1	c.820C>T(exon10)	Nonsynonymous Mutation	p.Arg274Trp	AD	Yes	Pathogenic
P6	STAT1	c.820C>T(exon10)	Nonsynonymous Mutation	p.Arg274Trp	AD	Yes	Pathogenic
P7	STAT1	c.821G>A(exon10)	Nonsynonymous Mutation	p.Arg274Gln	–	Yes	Pathogenic
P8	CD40LG	c.634_635del (exon5)	Frameshift Mutation	p.His212fs18	novel mutation	No	Pathogenic
P9	CD40LG	c.619_620del (exon5)	Frameshift Mutation	p.Arg207SerfsTer23	novel mutation	No	Pathogenic
P10	STAT3	c.1673G>T//WT c.1674C>T//WT (exon19)	Nonsynonymous Mutation	p.Gly558Val	novel mutation	No	Likely pathogenic
P11	CARD9	c.1144C>T(exon8) c.658G>C(exon5)	Nonsynonymous Mutation	p.Arg382Cys p.Ala220Pro	AD	No	Uncertain
P12	IL7R	c.355dupA(exon3)	Nonsynonymous Mutation	p.Ile121AsnfsTer8	–	No	Uncertain
P13	LYST	c.8368A>C(exon32) c.281C>T(exon4)	Nonsynonymous Mutation	p.Lys2790Gln p.Thr94Ile	AR	No	Likely pathogenic
P14	IL2RG	c.217A>C(exon2)	Nonsynonymous Mutation	p.Thr73Pro	XLR	No	Uncertain
P15	IL2RG	c.359del(exon3)	Frameshift Mutation	p.Lys120ArgfsTer27	XLR	No	Pathogenic
P16	KRAS	c.35G>C(exon2)	Nonsynonymous Mutation	p.Gly12Ala	novel mutation	No	Pathogenic
P17	DOCK8	c.1927C>G(exon17)	Nonsynonymous Mutation	p.His643Asp	–	No	Uncertain
P18	PLCG2	c.693-3del(intron8)	Deletion mutation	p.?	AD	No	Uncertain

Means the parents’ DNA was not available, the inheritance pattern could not be analyzed.

### 3.4 Treatment

9 patients with median APACHE II score of 17 (IQR:15.5-23) had to received advanced life support in ICU, including mechanical ventilatory support (n=9/18), continuous renal replacement therapy (CRRT; n=2/18) and plasma exchange (n=3/18). One patient died due to clinical deterioration before diagnosis of talaromycosis and conduction of anti-fungal treatment. A total of 17 patients received antifungal therapy. Detailed therapeutic medication and response were displayed in **Table 4**. Initial anti-fungal treatment regimens included: voriconazole (n=10), amphotericin B (n=2), itraconazole (n=2), fluconazole (n=1). 2 patients with severe clinical conditions received combination therapy with amphotericin B and voriconazole. The median duration of initial antifungal treatment was 14 days (IQR: 8-24). After the initial treatment, 15 surviving patients continued to receive voriconazole or itraconazole as maintenance therapy. The median duration of antifungal treatment was 16 months (IQR: 0.8-72.5 months). Most of the patients received antibiotic treatment as co-infected with bacteria, the common antibiotic regimens included meropenem (n=11/18) and piperacillin-tazobactam (n=10/18). Immunoenhancing therapy with IVIg and thymosin was administered to 12 patients, and 3 patients continued to regularly receive IVIg maintenance therapy until the end of the study. In addition, one SCID (IEI: *IL2RG* deficiency) patient underwent HSCT at an external hospital after 4 months of enrollment, and one patient (IEI: *CD40L* deficiency) underwent HSCT at an external hospital after 5 years of enrollment due to recurrent talaromycosis. The treatment regimens are summarized in **Table 1**.

**Table 4:** Overview of antifungal treatment effectiveness.

	Induction therapy	Maintenance therapy	Response in 2w	Relapse in 2w	Live period in 2w	Response in 12w	Relapse in 12w	Live period in 12w	Response in 24w	Relapse in 24w	Live period in 24w
P1	Voriconazole	Voriconazole	Partial	No	2w	Partial relief	No	12w	Complete	No	24w
P2	Voriconazole	–	Fail (died)	No	3d	–	–	–	–	–	–
P3	Amphotericin B +Voriconazole	Voriconazole	Partial	No	2w	Fail (died)	No	6w	–	–	–
P4	Amphotericin B	Itraconazole	Partial	No	2w	Partial	No	12w	Partial	No	24w
P5	Amphotericin B	–	Fail (died)	–	2d	–	–	–	–	–	–
P6	Fluconazole	Itraconazole	Partial	No	2w	Partial	No	12w	Partial	No	24w
P7	–	–	Fail (died)	–	2d	–	–	–	–	–	–
P8	Itraconazole	Itraconazole	Partial	No	2w	Partial	No	12w	Complete	No	24w
P9	Voriconazole	Voriconazole	Partial	No	2w	Partial	No	12w	Complete	No	24w
P10	Voriconazole	Voriconazole	Partial	No	2w	Partial	No	12w	Complete	No	24w
P11	Voriconazole	Voriconazole	Partial	No	2w	Partial	No	12w	Partial	No	24w
P12	Voriconazole	Voriconazole	Partial	No	2w	Fail (died)	No	18d	–	–	–
P13	Voriconazole	Voriconazole	Partial relief	No	2w	Partial	No	12w	Complete	No	24w
P14	Voriconazole	Voriconazole	Partial relief	No	2w	Partial	No	12w	Fail (died)	No	14w
P15	Amphotericin B +Voriconazole	Posaconazole	Partial relief	No	2w	Partial	No	12w	Partial	No	24w
P16	Voriconazole	Voriconazole	Partial relief	No	2w	Partial	No	12w	Fail (relapse)	Yes	24w
P17	Voriconazole	Voriconazole	Partial relief	No	2w	Partial	No	12w	Partial	No	24w
P18	Itraconazole	Itraconazole	Partial relief	No	2w	Partial	No	12w	Complete	No	24w

### 3.5 Outcome and long-term prognosis

The median follow-up duration was 27 months (IQR: 4-79.7 months). Briefly, 3 patients died within 2 weeks of diagnose, and other 3 patients died within 24 weeks, including 4 patients with STAT1-GOF and 2 patients with SCID. The information of IEI-related genetic defect was summarized in **Table 5**. One *CARD9* deficiency patient was lost to follow-up after one year, and 1 *KRAS* mutation patient died due to leukemia after 2 years. Therefore, the overall mortality rate was 41.2% in this cohort, and the mortality rate attributable to talaromycosis was 33.3%. The median duration from diagnosis of talaromycosis to death was 16 days (IQR:2–46 days).

**Table 5:** Information summarized by IEI-related genetic defects.

IEI genetic defect (n)	Novel/reported	Key immunological feature (from Table 2)	Severe complications (ICU, sepsis, HLH)	Relapse	Death (attributable to talaromycosis)	Long-term outcome
STAT1-GOF (7)	Reported	↓NK, ↓CD4, ↓IgG	6/7	1/7 (14%)	4/7 (57%) – 3 early deaths	1 longest survival (>20y on therapy), 2 stable on therapy
SCID (IL2RG/IL7R) (3)	IL2RG reported, IL7R novel	↓lymphocytes, ↓Ig	3/3	0/3	2/3 (67%) – both early deaths	1 alive post-HSCT (stable on therapy)
CD40L deficiency (2)	Novel mutations	↓IgG, ↓NK	1/2	0/2	0/2	Both stable on therapy (1 post-HSCT)
STAT3-LOF (1)	Novel	–	0/1	0/1	0/1	Relapse-free survival (no antifungal)
CARD9 (1)	Reported	–	1/1	Unknown (lost to Follow up)	Unknown (lost to Follow up)	Lost to follow-up at 1 year
LYST (CHS) (1)	Novel	–	0/1	0/1	0/1	Relapse-free survival (8 years)
DOCK8 (1)	Novel	–	0/1	0/1	0/1	Stable on therapy
PLCG2 (1)	Novel	–	0/1	0/1	0/1	Relapse-free survival (9 months)
KRAS (1)	Novel	–	1/1	0/1	1/1 (leukemia, not talaromycosis)	Died of leukemia

The risk of relapse after discontinuing antifungal therapy was concerned. 3 patients experienced relapsed after sustained antifungal therapy at 4, 6, and 14 months respectively, and then they were re-treated with antifungal drugs. Thus, in the research analysis period, 4 patients achieved relapse-free survival without antifungal treatment. The other patients were stable who maintenance antifungal therapy (One survived for more than 20 years, the longest recorded survival now). Until last follow-up, their relapse-free survival periods lasted for 8 years (IEI: *LYST* deficiency), 4 years (IEI: STAT1-GOF), 9 months (IEI: *PLCG2* deficiency), and 6 months (IEI: STAT3-LOF), respectively. Immune boosting therapy were given to the follow-up patients, although they might affect by bacteria or virus infection, all of them achieved long-term survival after treatment. Notably, though 2 children accepted HSCT could be challenged with infections from various types of pathogens, their prognosis were favorable.

## 4 Discussion

Talaromycosis in children is rare, and most pediatric patients are HIV-uninfected. In this 13-year cohort study, the rate of pediatric patients in HIV-uninfected talaromycosis population is accounting for 7%. Similar to previous studies, the clinical characteristics of pediatric talaromycosis in the study are atypical in the early stages of the disease (10, 11). 61.1% of patients initially were misdiagnosed with bronchial pneumonia or tuberculosis. Delayed treatment led to half of the children suffered from severe complications and had to received advanced life support in ICU. Our experience implied early diagnosis and prompt antifungal are essential for life saving. Moreover, pediatrician in epidemic areas should be aware of T. marneffei infection for children in ICU with unclear infection. As children probably already entered ICU before diagnosis, identification critical condition then given suitable life support is important to reduce the case fatality rate in children with talaromycosis.

IEI is a sort of disease caused by monogenic mutations, resulting in dysfunctions in immunity, and present vulnerable to infectious disease, autoimmune diseases, autoinflammatory diseases, and malignancies (6). The incidence rate of IEI ranges from 1‰ to 5‰, while cases of children with talaromycosis and IEI are less than 100 (12, 13). Studies have shown that 44.5% of children with talaromycosis have concomitant IEI. However, the true proportion may be even higher due to a significant number of affected children not undergoing IEI assessment (14). In the study, 100% children conduced WES were IEI, which indicated that in HIV-uninfected pediatric patients, talaromycosis serves as an early warning indicator for specific IEI, and genetic screening is necessary to identify underlying single-gene defects.

Till now, 11 types of IEI have been reported with talaromycosis, including STAT3-LOF, STAT1-GOF, *CD40L*, *IL2RG*, *IFNGR1*, *IL12RB1*, *CARD9*, *COPA*, *ADA*, *RELB*, and *NFKB2* deficiency (15). In our study, we identified novel genetic defects associated with talaromycosis, including *IL7R*, *LYST*, *PLCG2*, *DOCK8*, and *KRAS* deficiency. These findings expand the spectrum of IEI underlying talaromycosis. In literature, *IL7R*, *LYST*, *PLCG2*, *DOCK8* regulate the function and differentiation of T cells, B cells, and NK cells, who mainly involved in adaptive immunity. *KRAS* mutations frequently occurred in multiple human tumors, inhibited T-cell infiltration and recruited suppressive immune cells, thereby creating an immunosuppressive microenvironment (16). IEI children with talaromycosis exhibited decreased lymphocytes, NK cells, and immunoglobulin levels. The mortality of novel and reported IEIs are close, displaying the collaborating roles of innate and adaptive immunity in combating talaromycosis. However, the roles of most IEI genetic defects in fungal infections remains to be clarified, and intersection between gene mutations and immune responses against microbial infections should be further illustrated. As the follow up continues in the future and more children with talaromycosis and IEI will be enrolled, we believe a broaden IEI spectrum and the unclear mechanism could be discovered.

In our cohort, 7 patients (41.2%) died during follow-up, including 4 patients with STAT1-GOF, 2 patients with SCID (1 *IL2RG* and 1 *IL7R* deficiency), and 1 patient died for leukemia with *KRAS* mutations. These data demonstrated that specific IEI genetic defects, such as SCID and STAT1-GOF, require careful attention during the whole therapeutic period and long-term follow-up. Nevertheless, the patient with the longest survival was a STAT1-GOF patient who has survived for over 20 years to date. To our knowledge, this represents the longest documented survival in the literature for this specific type of IEI (17, 18). Extra attention should be paid to patients with SCID and STAT1-GOF for their high mortality, potentially resulting in the hindered development of the core axis of T cells. Additionally, our study first showed died children exhibited significantly lower levels of GM-CSF, IFN-γ, and IL-12p70 than those alive, indicating the crucial function of IL12/IFN-γ axis in the talaromycosis. Early gene sequencing and cytokines evaluation could contribute to talaromycosis patient stratification and prognosis prediction.

Our study provides a descriptive genotype–outcome matrix for talaromycosis in children with IEI (**Tables 4**, **5**). STAT1−GOF was the most common defect (7/18) and was associated with both the highest mortality (57%) and the longest documented survival (>20 years). This paradoxical finding may reflect variable expressivity or the influence of co−morbidities and treatment intensity. SCID (IL2RG/IL7R) uniformly led to severe, often fatal disease unless rescued by HSCT, consistent with the critical role of T−cell immunity in controlling Talaromyces. Notably, patients with novel defects affecting adaptive immunity (LYST, DOCK8, PLCG2) achieved relapse−free survival or stable disease with antifungal maintenance, suggesting that some residual immune function may permit long−term control. The single CARD9 deficiency patient, despite severe acute disease, was lost to follow−up; CARD9 is known to be important for antifungal immunity, but long−term outcome in this context remains unclear. These observations, while hypothesis−generating, highlight the need for genotype−guided risk stratification and personalized management, including early consideration of HSCT for severe combined immunodeficiencies and careful monitoring of STAT1−GOF patients who may survive for decades with appropriate antifungal and immune support.

It could be concluded form this cohort, anti-fungal therapy laid the foundation for long-time survival of children with talaromycosis and IEI, while immune boosting therapy as well as advanced life support helped them pull through the crisis from infection and damaged immune system. Immune boost therapy is a novel therapeutic intervention for fungal infection (19, 20). In this study, compared to the baseline, most peripheral immunological did not exhibit a significant change after treatment except IgG, displaying a lasting immunocompromised state in IEI children. Notably, intravenous immunoglobulin could help to increase the IgG level, which is consistent with previous study (21). HSCT was reported to help children with IEI attain T and B cell reconstitution (22, 23). However, the safety and efficacy of HSCT for children with IEI and talaromycosis remain unclear. We first disclosed the long-time follow up of this specific type of patients who achieved prolong survival. Our study provided valuable experience for the management of children with talaromycosis as well as IEI.

There are several limitations should be considered. As a multi-center cohort study for rare disease, the enrolled population is small. Moreover, only baseline cytokines were displayed, more follow-up data were being collected. In summary, this study uncovered the potential association between clinical characteristics and long-term prognosis of pediatric patients with talaromycosis and IEI. These findings preliminarily provided a foundation for future personalized diagnosis and management as well as guideline development.

## Data Availability

The raw data supporting the conclusions of this article will be made available by the authors, without undue reservation.
